# Supplementary material for: Spatially and cell-type resolved quantitative proteomic atlas of healthy human skin
Source: Nat Commun. 2020 Nov 5;11:5587. doi: 10.1038/s41467-020-19383-8 (PMC7645789; doi:10.1038/s41467-020-19383-8)
Supplement: Supplementary file 1 — Supplementary Information [file 41467_2020_19383_MOESM1_ESM.pdf]

Supplementary Information

Spatially and cell-type resolved quantitative proteomic atlas of healthy human skin

Beatrice Dyring-Andersen[1,2,3], Marianne Bengtson Løvendorf[4], Fabian Coscia[1], Alberto Santos[1], Line Bruun Pilgaard Møller[1], Ana R. Colaço[1], Lili Niu[1], Michael Bzorek[5], Sophia Doll[6], Jørgen Lock Andersen[7], Rachael A. Clark[2], Lone Skov[8], Marcel B.M. Teunissen[9], Matthias Mann[1,6*]

[1]Novo Nordisk Foundation (NNF) Center for Protein Research, Faculty of Health and Medical Sciences, University of Copenhagen, Denmark
[2]Department of Dermatology, Brigham and Women's Hospital and Harvard Medical School, Boston, US
[3]Leo Foundation Skin Immunology Research Center, Faculty of Health and Medical Sciences, University of Copenhagen, Denmark
[4]Center for RNA Medicine, Department of Clinical Medicine, Aalborg University, Denmark
[5]Department of Surgical Pathology, Zealand University Hospital, Denmark
[6]Department of Proteomics and Signal Transduction, Max Planck Institute of Biochemistry, Martinsried, Germany
[7]Department of Plastic and Breast Surgery, Zealand University Hospital, Denmark
[8]Department of Dermatology and Allergology, Herlev and Gentofte Hospital, University of Copenhagen, Denmark
[9]Department of Dermatology, Amsterdam University Medical Centers, location AMC, Amsterdam, Netherlands

Supplementary Figure 1

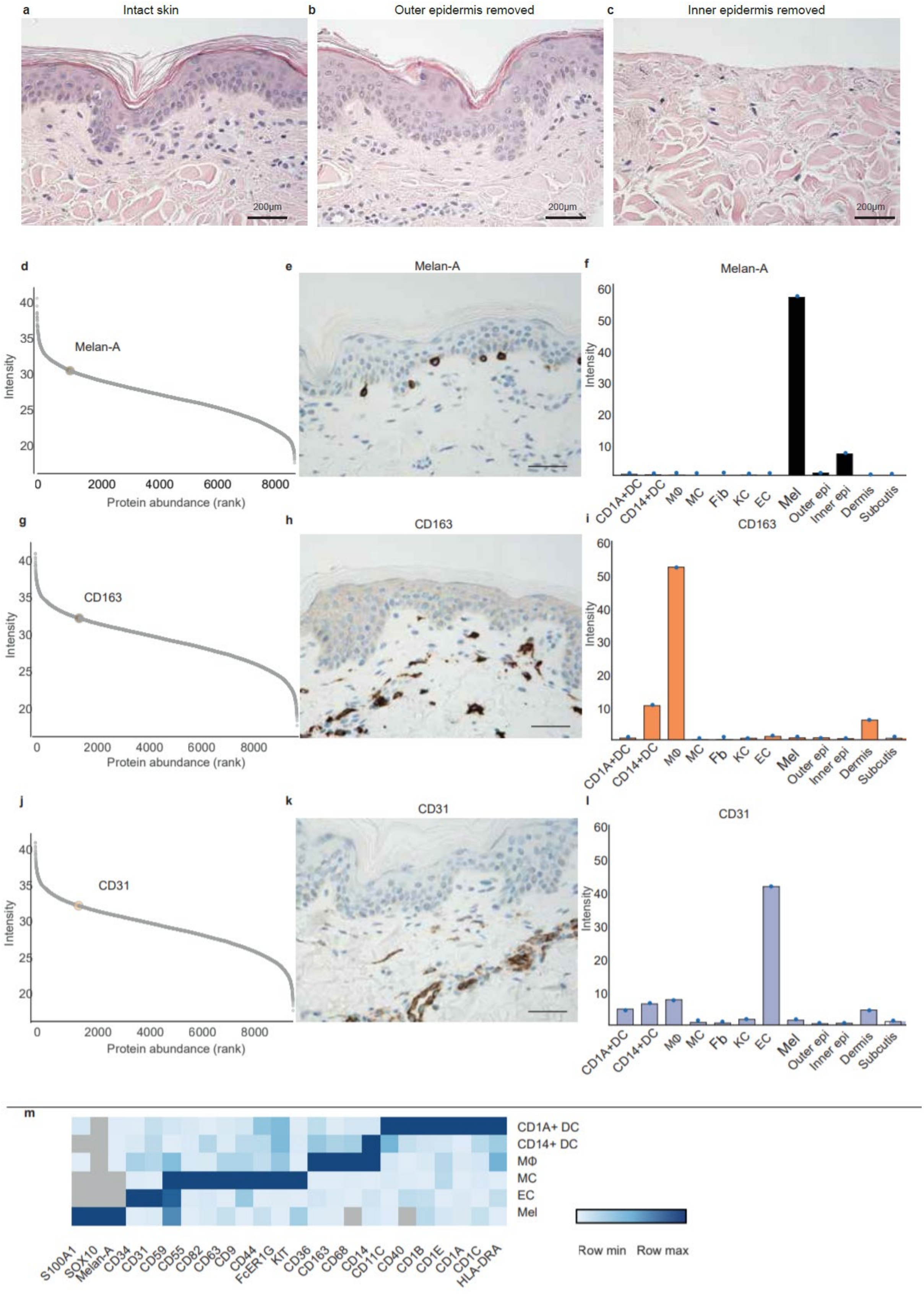

Supplementary Fig. 1. Histological verification and characteristic cell subset markers. Representative hematoxylin and eosin (HE) stainings of healthy donor skin before a) and after b) curettage of outer epidermis and c) HE staining of skin after curettage of inner epidermis down to the basal lamina. d) Estimated protein abundance vs protein rank of Melan-A in FACS sorted melanocytes (Mel). e) Immunohistochemical staining of Melan-A in healthy skin. f) Estimated abundance of Melan-A across skin layers and skin-derived cells. g) Estimated protein abundance vs protein rank of CD163 in FACS sorted macrophages (MΦ). h) Immunohistochemical staining of CD163. i) Estimated abundance of CD163 across skin layers and skin-derived cells. j) Estimated protein abundance vs protein rank of CD31 in FACS sorted endothelial cells (EC). k) Immunohistochemical staining of CD31. l) Estimated abundance of CD31 across skin layers and skin-derived cells. m) Heatmap visualizing the protein expression of characteristic markers for cellular subsets. $CD1A^+$ DC; dermal dendritic cells, CD14+ DC; CD14+ dendritic cells, MΦ; macrophages, MC; mast cells, EC; endothelial cells, Mel; melanocytes, Fib; fibroblast, KC; keratinocytes. All immunohistochemical stainings are representative images of N=5. Scale bars: 50µm. All barplots show estimated intensity based on one library of pooled samples of skin layers (outer epi, inner epi, dermis, subcutis; N=5) and cell subsets (DC; $CD1A^+$dendritic cells (N=3), CD14+ dendritic cells (N=3), MΦ; macrophages (N=4), MC; mast cells (N=6), Fib; fibroblasts (N=5), KC; keratinocytes (N=5), EC; endothelial cells (N=4), Mel; melanocytes (N=5).

Supplementary Figure 2

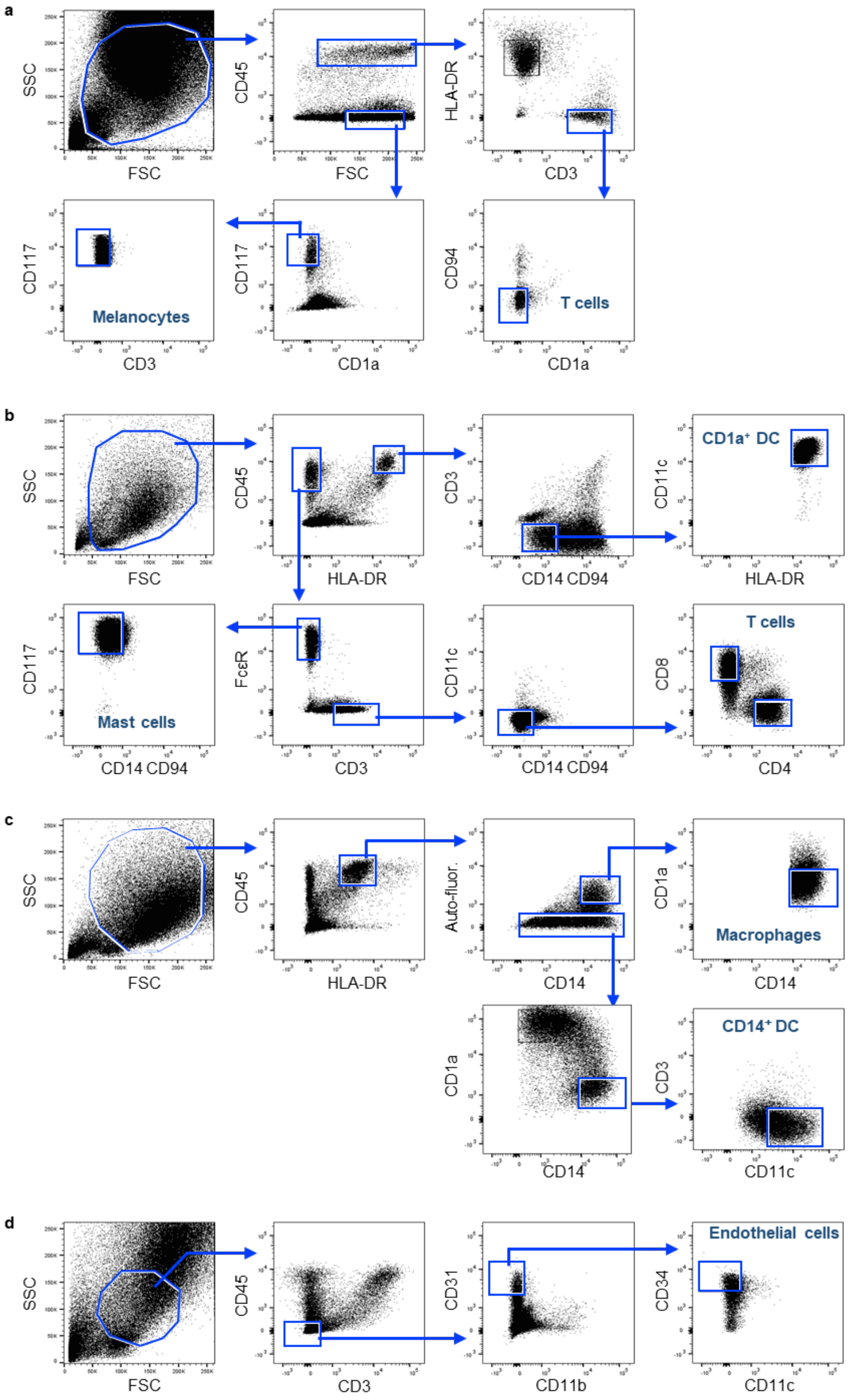

Supplementary Fig. 2. Sorting strategy for isolation of specific cell subsets from the skin. a) Sorting strategy for isolation of epidermal T cells (ET) and melanocytes (Mel). b) Sorting strategy for isolation of mast cells (MC), $CD1a^+$ dendritic cells (DC) and dermal T cells (DT). c) Sorting strategy for isolation of macrophages (MΦ) and $CD14^+$ dendritic cells ($CD14^+$ DC) from dermis. d) Sorting strategy for isolation of endothelial cells (EC).

Supplementary Figure 3

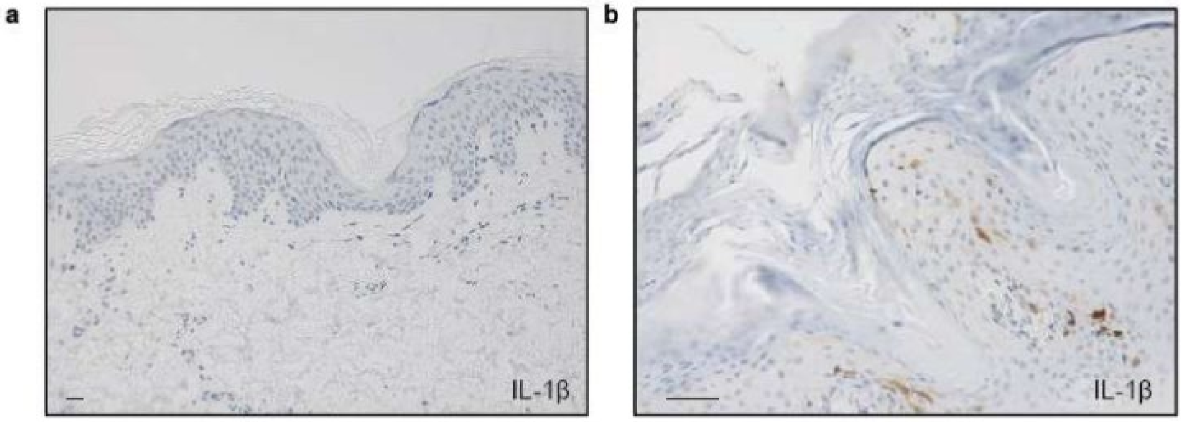

Supplementary Fig. 3. IL-1ß expression in the skin. Representative immunohistochemical images of IL-1β in a) healthy skin (N=3) (not detectable) and b) psoriatic skin (N=3) where it was found in the macrophages and dendritic cells in the dermis. Scale bar: 50µm.
