# Supplementary material for: Spatially and cell-type resolved quantitative proteomic atlas of healthy human skin
Source: Nat Commun. 2020 Nov 5;11:5587. doi: 10.1038/s41467-020-19383-8 (PMC7645789; doi:10.1038/s41467-020-19383-8)
Supplement: Supplementary file 3 — Description of Additional Supplemtary Files [file 41467_2020_19383_MOESM3_ESM.docx]

File Name: Supplementary Data 1

Description: MS-Intensity (IBAQ) of the 10,701 protein groups detected in the data-dependent acquisition mode (DDA).

File Name: Supplementary Data 2

Description: Relative expression levels (log2) of important structural and immunological proteins across skin layers and in primary keratinocytes and fibroblasts based on data-dependent acquisition (DDA) data.

File Name: Supplementary Data 3

Description: Protein profiles with reference profile CD248 – Fibroblasts.

File Name: Supplementary Data 4

Description: Protein profiles with reference profile KLK10 – Keratinocytes.

File Name: Supplementary Data 5

Description: Standard ANOVA analysis between cellular proteomes of the FACS sorted cells (FDR < 0.05)

File Name: Supplementary Data 6

Description: List of differentially expressed proteins (posthoc pairwise t-test analysis) across cellular subsets.

File Name: Supplementary Data 7

Description: List of kinases among differentially expressed proteins in cellular subsets (ANOVA, FDR < 0.01; fold-change>2) cross ref to Molecular Function GO:0016301.

File Name: Supplementary Data 8

Description: List of ligases among differentially expressed proteins in cellular subsets (ANOVA, FDR < 0.01; fold-change>2) cross ref to Molecular Function GO:0061630.

File Name: Supplementary Data 9

Description: Protein profiles for dendritic cells (DC) with reference profile CD1C.

File Name: Supplementary Data 10

Description: Protein profiles for macrophages (MΦ) with reference profile CD163.

File Name: Supplementary Data 11

Description: Protein profiles for mast cells (MC) with reference profile CPA3.

File Name: Supplementary Data 12

Description: Protein profiles for epidermal (ET) and dermal T cells (DT) with reference profile CD3d.

File Name: Supplementary Data 13

Description: List of 260 differentially expressed proteins between epidermal T cells (ET) and dermal T cells (DT).

File Name: Supplementary Data 14

Description: Protein profiles for endothelial cells (EC) with reference profile CDH5.

File Name: Supplementary Data 15

Description: Protein profiles for melanocytes (Mel) with reference profile Melan-A.

File Name: Supplementary Data 16

Description: List of antibodies used in this study.

File Name: Supplementary Data 17

Description: Courtesy of Human Protein Atlas, www.proteinatlas.org.

File Name: Supplementary Data 18

Description: Overview of fractions for the data-dependent acquisition (DDA) data.
