# Supplementary material for: Spatially and cell-type resolved quantitative proteomic atlas of healthy human skin
Source: Nat Commun. 2020 Nov 5;11:5587. doi: 10.1038/s41467-020-19383-8 (PMC7645789; doi:10.1038/s41467-020-19383-8)
Supplement: Supplementary file 22 — Reporting Summary [file 41467_2020_19383_MOESM22_ESM.pdf]

nature research

Corresponding author(s): Matthias Mann

Last updated by author(s): Aug 17, 2020

Reporting Summary

Nature Research wishes to improve the reproducibility of the work that we publish. This form provides structure for consistency and transparency in reporting. For further information on Nature Research policies, see our Editorial Policies and the Editorial Policy Checklist.

Statistics

For all statistical analyses, confirm that the following items are present in the figure legend, table legend, main text, or Methods section.

n/a	Confirmed	
☐	☒	The exact sample size (n) for each experimental group/condition, given as a discrete number and unit of measurement
☐	☒	A statement on whether measurements were taken from distinct samples or whether the same sample was measured repeatedly
☐	☒	The statistical test(s) used AND whether they are one- or two-sided<br>*Only common tests should be described solely by name; describe more complex techniques in the Methods section.*
☒	☐	A description of all covariates tested
☐	☒	A description of any assumptions or corrections, such as tests of normality and adjustment for multiple comparisons
☐	☒	A full description of the statistical parameters including central tendency (e.g. means) or other basic estimates (e.g. regression coefficient) AND variation (e.g. standard deviation) or associated estimates of uncertainty (e.g. confidence intervals)
☐	☒	For null hypothesis testing, the test statistic (e.g. F, t, r) with confidence intervals, effect sizes, degrees of freedom and P value noted<br>*Give P values as exact values whenever suitable.*
☒	☐	For Bayesian analysis, information on the choice of priors and Markov chain Monte Carlo settings
☐	☒	For hierarchical and complex designs, identification of the appropriate level for tests and full reporting of outcomes
☒	☐	Estimates of effect sizes (e.g. Cohen's d, Pearson's r), indicating how they were calculated

Our web collection on statistics for biologists contains articles on many of the points above.

Software and code

Policy information about availability of computer code

Data collection	ODA raw files were processed in the MaxQuant environment (1.6.3.4). DIA raw files were analyzed using Spectronaut Pulsar X software (Biognosys, version 12.0.20491.17)
Data analysis	Perseus, the R framework (https://www.r-project.org/) and CKG's analytics core module (https://github.com/MannLabs/CKG)

For manuscripts utilizing custom algorithms or software that are central to the research but not yet described in published literature, software must be made available to editors and reviewers. We strongly encourage code deposition in a community repository (e.g. GitHub). See the Nature Research guidelines for submitting code & software for further information.

Data

Policy information about availability of data

All manuscripts must include a data availability statement. This statement should provide the following information, where applicable:

- Accession codes, unique identifiers, or web links for publicly available datasets
- A list of figures that have associated raw data
- A description of any restrictions on data availability

The mass spectrometry proteomics data have been deposited to the ProteomeXchange Consortium via the PRIDE partner repository with the dataset identifier PXD019909 (username: reviewer26347@ebi.ac.uk, password: gC4bRv4h during the review process)60.

Field-specific reporting

Please select the one below that is the best fit for your research. If you are not sure, read the appropriate sections before making your selection.

- [x] Life sciences
- [ ] Behavioural & social sciences
- [ ] Ecological, evolutionary & environmental sciences

For a reference copy of the document with all sections, see nature.com/documents/nr-reporting-summary-flat.pdf

Life sciences study design

All studies must disclose on these points even when the disclosure is negative.

Sample size	No sample size calculation was performed. We have included samples from 32 donors enabling us to analyse at least triplicates for all samples. Most data on skin layer and cellular subsets are on N=5.
Data exclusions	We haven't excluded data.
Replication	The mass spectrometry experiments described in this study were performed independently on the same mass spectrometer
Randomization	Donors were anonymous and the project included one group. No randomization took place during processing or analyses of tissue and cells. The analyses performed were complex and took months to perform and there was a need to keep a strict overview of all samples, passages, fractions and LC MS/MS runs.
Blinding	Investigators were not blinded during processing or analyses in this study to keep a strict overview of samples, passages, fractions and LC MS/MS runs.

Reporting for specific materials, systems and methods

We require information from authors about some types of materials, experimental systems and methods used in many studies. Here, indicate whether each material, system or method listed is relevant to your study. If you are not sure if a list item applies to your research, read the appropriate section before selecting a response.

Materials & experimental systems

n/a	Involved in the study	
[ ]	[x]	Antibodies
[x]	[ ]	Eukaryotic cell lines
[x]	[ ]	Palaeontology and archaeology
[x]	[ ]	Animals and other organisms
[ ]	[x]	Human research participants
[ ]	[x]	Clinical data
[x]	[ ]	Dual use research of concern

Methods

n/a	Involved in the study	
[x]	[ ]	ChIP-seq
[ ]	[x]	Flow cytometry
[x]	[ ]	MRI-based neuroimaging

Antibodies

Antibodies used

Flowcytometry
CDla-FITC BD Pharmingen #555806 1:100
CD1a-BV421 BioLegend #300128 1:100
CD3-PE-Cy7 BioLegend #344816 1:100
CD3-PE-CF594 BD Biosciences #562280 1:200
CD3-BV605 BioLegend #317322 1:100
CD4-Dazzle594 BioLegend #317448 1:200
CD8-PE-Cy7 BD Pharmingen #557746 1:100
CDllb-PE Immunotech #PN IM2581 1:100
CDllc-APC BD Biosciences #333144 1:100
CD14-FITC BD Biosciences #345784 1:100
CD14-Dazzle594 BioLegend #325634 1:200
CD31-FITC BioLegend #303104 1:100
CD34-PE-Cy7 BioLegend #343516 1:100
CD45-APC-Cy7 BD Biosciences #348815 1:200
CD94-FITC BD Pharmingen #555888 1:100
CD94-APC BioLegend #305508 1:100
CDll 7-PE-Cy5.5 Beckman Coulter #A66333 1:100
FcERI-BV421 BioLegend #334624 1:100

HLA-DR-PE BD Pharmingen #555812 1:100
Immunohistochemical stainings
CD31 JC/70A 20069553 Mouse Agilent/Dako, Glostrup, Denmark GA610 TRS High pHI Ready-to-use
CD163 MRQ-26 000028057 Mouse Cell Marque, Rocklin, CA,USA 163M-16 TRS High pH 1:25
MART1 EP43 20040201 Rabbit Cell Marque, Rocklin, CA,USA AC-0041 TRS High pH 1:100
RR3 Collagen III Polyclonal 45324 Rabbit LSBio, Seattle, WA, USA LS-B693 TRS Low pH + Pepsin2 1:500 III- Polyclonal QM1018101 Goat R&D Systems, Minneapolis, MN, USA AF-201-NA TRS High pH 1:50 RR
CKIO DE-KIO 20052315 Mouse Agilent/Dako, Glostrup, Denmark M7002 TRS High pH 1:50
CK14 SP53 150127LVH Rabbit Abeam, Cambridge, UK Ab119695 TRS High pH 1:200

Validation

IHC antibodies are all validated at the Pathology department, Zealand Hospital. CD31, CD163, MARTl/Melan A are calibrated using the External Quality Assurance program NordiQC. CDlb was tested on multiple tissues for validation and quality purposes. All the fluorescently-labeled antibodies are commercially available and validated for the specificity to the indicated human antigens and application for flow cytometry, as stated in the manufacturer's catalogue and website.

Human research participants

Policy information about studies involving human research participants

Population characteristics

Discarded human skin tissue was obtained (anonymopusly and without consent) from healthy donors (aged between 18 and 65 years) undergoing corrective surgery of the breast or abdomen.

Recruitment

Skin from surgeries was handed over to the authors of this paper directly after surgery. None of the authors were involved with surgeries or planning hereof.

Ethics oversight

The study was carried out in agreement with the Danish and Dutch law (Medical Research Involving Human Subjects Act), in accordance with the guidelines of the Medical Ethics Review Committees of the involved institutes and following the Declaration of Helsinki principles.
Discarded skin tissues from corrective surgery of the breast or abdomen were anonymized prior to providing them to the authors of the paper, who were not involved in the surgeries during which the tissues were collected. According to the Danish and Dutch law, researchers are allowed to use discarded anonymous tissue without patient consent.
Human peripheral blood was obtained from healthy adult volunteers (independent from the skin donors) via a vene puncture after written informed consent using an approved protocol by the Ethics Committee of the Capital Region of Denmark (H-3-2014-123 version 1 from 30 07 2018) and complying with the ethical regulations for work with human participants.

Note that full information on the approval of the study protocol must also be provided in the manuscript.

Clinical data

Policy information about clinical studies
All manuscripts should comply with the ICMJEguidelines for publication of clinical research and a completedCONSORT checklist must be included with all submissions.

Clinical trial registration

Not relevant to this study.

Study protocol

Not relevant to this study.

Data collection

Not relevant to this study.

Outcomes

Not relevant to this study.

Flow Cytometry

Plots

Confirm that:

- [x] The axis labels state the marker and fluorochrome used (e.g. CD4-FITC).
- [x] The axis scales are clearly visible. Include numbers along axes only for bottom left plot of group (a 'group' is an analysis of identical markers).
- [x] All plots are contour plots with outliers or pseudocolor plots.
- [x] A numerical value for number of cells or percentage (with statistics) is provided.

Methodology

Sample preparation

Skin: Sheets of 0.3–0.4 mm thickness were prepared with an electrodermatome and were treated overnight at 4°C with 0.2% (wt/vol) dispase II (Boehringer Mannheim, Mannheim, Germany) in PBS with 50 µg/ml gentamycin (Sigma, St. Louis, MO). Epidermis and dermis were separated by forceps, and in order to get fresh single cell suspensions, the epidermis was fragmented by scissors and incubated in 0.25% (wt/vol) trypsin in PBS for 30 min at 37°C, while the fragmented dermis was incubated in IMDM (Corning, cat. no. MT10016CV) with 0.4%(wt/vol) collagenase D (Sigma, cat. no. C0130-1G), 50 U/mL DNAse I (Sigma, cat. no. D4263-1VL) and 0.5% (vol/vol) FCS for 2–3 h at 37°C. After enzymatic digestion, the cell suspensions were sieved through 70-µm cell strainers (Falcon) to remove tissue debris, yielding single cells with a viability exceeding 97%.

	In order to sort epidermal T cells and melanocytes by flow cytometry, the cell surface of freshly-prepared epidermal cells were stained with antibodies.
Instrument	FACSAria cell-sorting equipment (BD Biosciences)
Software	BD FACSDiva v8, FlowJo vl0.4
Cell population abundance	The purity of the cells isolated by flow cytometry was determined in the same gate used for sorting and expressed as percentage of total lymphocytes in post-sort samples.
Gating strategy	ollowing CD45 positive gating, epidermal T cells were gated as CD3 positive and HLA-DR, CDla, and CD94 negative, whereas dermal T cells were gated as CD3 positive and HLA-DR, FcERI, CDllc, CD14 and CD94 negative, being either CD4 or CDS positive. Following CD45 positive gating and selection of HLA-DR positive cells, dermal CDla+ dendritic cells were gated as CDllc positive and CD3, CD14 and CD94 negative, whereas dermal CD14+ dendritic cells were gated as CDllc and CD14 positive and CD3, CDla and auto-fluorescent negative. Dermal macrophages were gated as auto-fluorescent and CD14 positive, following CD45 and HLA-DR positive gating. Dermal mast cells were gated as FcERI and CD117 positive and HLA-DR, CD3, CD14 and CD94 negative, following CD45 positive gating. Following CD45 negative gating, epidermal melanocytes were gated as CDll 7 positive and CD la, and CD3 negative, whereas dermal endothelial cells were gated as CD31 and CD34 positive and CD3, CDllb and CDllc negative. Details of the gating strategy are shown in Supplementary Figures 2a-d.

☒ Tick this box to confirm that a figure exemplifying the gating strategy is provided in the Supplementary Information.
